# Photopharmacology of Antimitotic Agents

**DOI:** 10.3390/ijms23105657

**Published:** 2022-05-18

**Authors:** Susanne Kirchner, Zbigniew Pianowski

**Affiliations:** 1 Institute of Organic Chemistry, Karlsruhe Institute of Technology, 76131 Karlsruhe, Germany; susanne.kirchner@kit.edu; 2 Institute of Biological and Chemical Systems–FMS, Karlsruhe Institute of Technology, 76344 Eggenstein-Leopoldshafen, Germany

**Keywords:** photopharmacology, microtubule-targeting agents, antimitotic agents, molecular photoswitches

## Abstract

Antimitotic agents such as the clinically approved vinca alkaloids, taxanes and epothilone can arrest cell growth during interphase and are therefore among the most important drugs available for treating cancer. These agents suppress microtubule dynamics and thus interfere with intracellular transport, inhibit cell proliferation and promote cell death. Because these drugs target biological processes that are essential to all cells, they face an additional challenge when compared to most other drug classes. General toxicity can limit the applicable dose and therefore reduce therapeutic benefits. Photopharmacology aims to avoid these side-effects by introducing compounds that can be applied globally to cells in their inactive form, then be selectively induced to bioactivity in targeted cells or tissue during a defined time window. This review discusses photoswitchable analogues of antimitotic agents that have been developed by combining different photoswitchable motifs with microtubule-stabilizing or microtubule-destabilizing agents.

## 1. Targeting Microtubule Dynamics

Tubulin is among the most studied targets for cytotoxicity. It builds microtubules, which are a major component of the eukaryotic cytoskeleton. This, in turn, is involved in essential biological processes including cellular motility, intracellular transport and cell division, which are so critical to cell survival that their inhibition almost inevitably leads to cell death. Although tubulin-targeting agents can affect all types of cells, many of them became potent therapeutics when selectively applied against cancer or inflammatory diseases [1,2].

**Figure 1:**
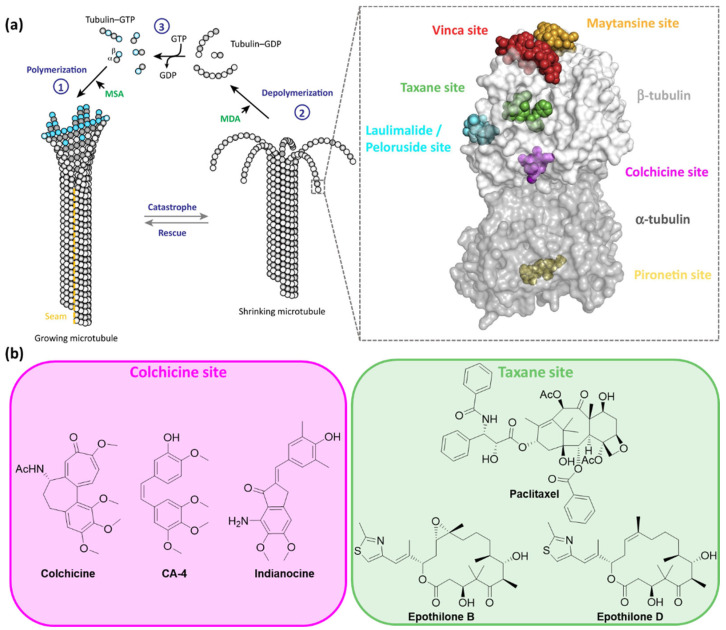
(**a**) The equilibrium of GTP-dependent assembly of α/β-tubulin heterodimers is referred to as ‘dynamic instability’. Microtubule stabilizing agents (MSAs) shift the equilibrium towards the microtubule (“1”—polymerization) and microtubule destabilizing agents (MDAs) towards tubulin heterodimers (“2”—depolymerization). The depolymerized fragments are activated (“3”—transesterification) for the following assembly process. Six binding sites of microtubule-targeting agents on tubulin have been identified and are highlighted in green (taxane site), cyan (laulimalide/peloruside site), magenta (colchicine site), slate (vinca site), orange (maytansine site) or yellow (pironetin site). (**b**) Photoswitchable analogues of microtubule destabilizing agents binding to the colchicine site and microtubule stabilizing agents binding to the taxane site have been developed. Adapted with permission from the ref. [2]. Copyrights (2018) Elsevier.

Microtubules are rigid hollow tube-like noncovalent polymers with a diameter of ∼25 nm that are composed of α/β-tubulin heterodimers. These heterodimers bind head-to-tail forming long strands called protofilaments and typically 13 protofilaments associate laterally to form microtubules (Figure 1). This parallel alignment leads to polar polymers with one more dynamic ‘plus end’ exposing β-tubulin subunits and the other less dynamic ‘minus end’ exposing α-tubulin subunits [3,4,5,6].

Microtubules form networks throughout the cell that are constantly remodeled through rapid growth and shrinkage at both ends by the addition and removal of tubulin heterodimers, a phenomenon that is referred to as ‘dynamic instability’ [7]. These dynamics enable microtubules to fulfil spatiotemporally distinct functions that require constant remodeling and are powered by GTP hydrolysis. Both subunits of the tubulin heterodimer can bind guanosine triphosphate (GTP) at the respective *N*-terminal regions. Whereas the GTP binding site of α-tubulin is hidden in the monomer–monomer interface of the heterodimer and has no GTPase activity, the binding site of β-tubulin is an exchangeable nucleotide-binding site that can bind either GDP or GTP. GTP-tubulin has the ability to assemble and polymerize whereas GDP-tubulin is more prone to depolymerization. Shortly after polymerization, GTP hydrolysis proceeds at β-tubulin initiated by the now adjacent α-tubulin, which provides an essential residue to the catalytic pocket [8]. Provided that the GTP-tubulin concentration is above a critical concentration, the growth proceeds more rapidly than the hydrolysis and a GTP cap is maintained at the growing end [9]. However, once the GTP-tubulin concentration drops below a threshold level and the GTP-cap is lost, protofilaments start to peel off from the microtubule body and dissociate into small oligomers and α/β-tubulin heterodimers. This transition from a phase of growing to dissociation is called ‘catastrophe’ and the reverse process is referred to as ‘rescue’. The kinetics of these processes are regulated by microtubule-associated proteins (MAPs) [2,10].

Microtubule-targeting agents are usually grouped based on their mode of action at high concentrations on the microtubule polymer mass. Microtubule-stabilizing agents prevent necessary depolymerization, whereas microtubule-destabilizing agents remove necessary microtubule polymers. However, at lower concentrations, both types of agents do not primarily affect the bulk of the microtubule polymer but suppress microtubule dynamics. As a result, these compounds disturb mitosis and induce an arrest of cells during the G_2_/M phase of the cell cycle (Figure 1) [1,2,11,12].

Several hundred microtubule-targeting compounds that are able to arrest mitosis have been reported. Thanks to structural biology, six distinct ligand-binding sites on the α/β-tubulin heterodimer have been identified and characterized. They are named after the most prominent agent or class of agents that bind to this region: laulimalide/peloruside, maytansine, taxane/epothilone, pironetin, vinca alkaloid and colchicine sites [2].

The nonspecifity of microtubule-targeting agents can cause systemic side-effects that significantly limit the doses that can be applied in chemotherapy or even prevent the approval of these compounds as therapeutics. Myelosuppression is frequently observed, and neutropenia is a very common and severe side effect that limits the applicable dose and can impair the therapeutic value of these drugs [1]. To address these limitations, photoswitchable analogues of microtubule-targeting agents have been developed to enable photoinduced activation at the tumor site by irradiation of affected tissue. These photopharmaceuticals can additionally allow the investigation of the dynamics of the microtubule cytoskeleton in non-invasive studies.

## 2. Practical Considerations

The implementation of a photoresponsive motif into the structure of a drug or the incorporation of the drug into a photoresponsive drug delivery system can enable the control of its pharmacokinetic and pharmacodynamic properties by irradiation. Light as a trigger of drug activity has the great advantage of being tunable in three dimensions: its location, its timing and its intensity [13].

**Figure 2:**
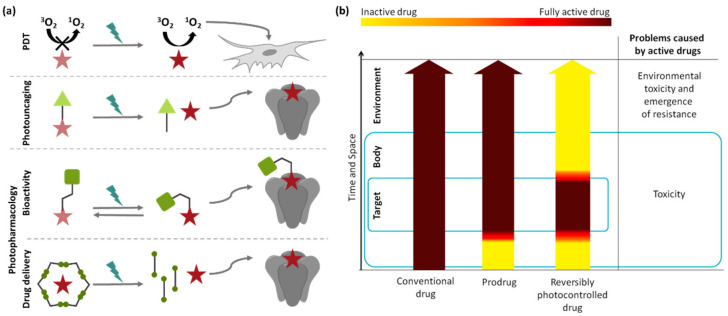
Basic principles of light-dependent pharmacology. (**a**) In photodynamic therapy (PDT), chromophores are applied that generate highly toxic, non-specific singlet oxygen (^1^O_2_) upon relaxation from a light-induced excited state. Irreversible photouncaging uses photoresponsive prodrugs that release active drugs through light-induced cleavage of a covalent bond and photopharmacology uses photoswitchable drugs that can be reversibly activated with light. Additionally, photoswitchable drug delivery systems enabling light-triggered drug release have been developed. (**b**) Comparison of drug activity over time and space during treatment with conventional drugs, photouncagable prodrugs and reversibly photoswitchable drugs. Photopharmacology aims to prevent adverse effect and environmental buildup by enabling light-triggered activation selectively at the target site [16,17]. Reprinted with permission from the ref. [16]. Copyrights (2014) American Chemical Society.

To enable light-dependent pharmacology, three approaches have been investigated: photodynamic therapy (PDT), photouncaging and photopharmacology (Figure 2). PDT relies on photosensitizers, which, upon irradiation, generate highly cytotoxic reactive oxygen species [14]. The drawback of this method is the general toxicity and lack of specificity in its activity. This is not the case in photouncaging, where a photoresponsive prodrug is applied and the active drug released through light-induced cleavage of a covalent bond [15]. The disadvantages of photouncaging include, for example, the irreversibility of the photoactivation as well as the formation of byproducts, or enzymatic hydrolysis. The concept of photopharmacology refers to the introduction of a photoswitchable moiety into the molecular scaffold of a bioactive compound to generate a pharmaceutical with significantly higher activity in one structural state. This, in turn, allows for the reversible control of bioactivity while maintaining specificity [16].

**Figure 3:**
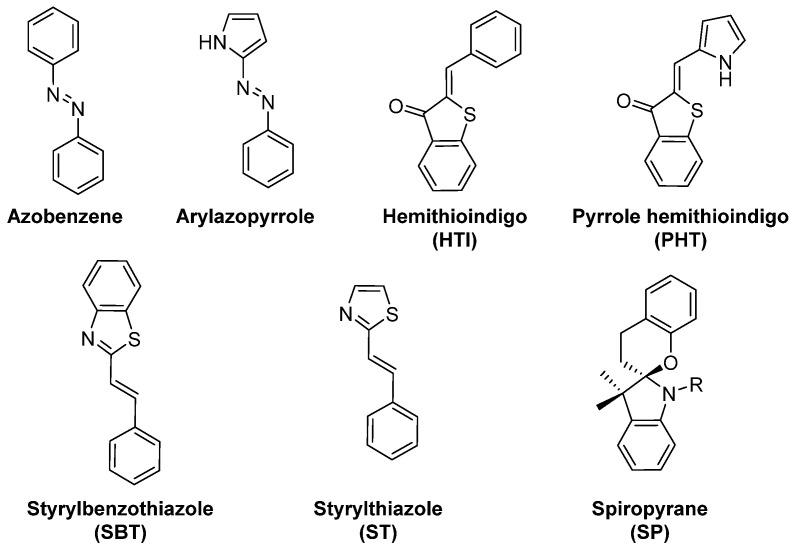
Overview of molecular photoswitches that have been applied to design photoswitchable microtubule-targeting agents.

When designing photoresponsive drugs, one is bound by several factors concerning the photophysical properties of the applied photoswitchable motif (examples depicted on the Figure 3), as well as the pharmacological properties of the resulting compound.

### 2.1. Photophysical Properties

The applicability of light is limited by two aspects: the potential phototoxicity of UV-light and the wavelength-dependent amount of light that can penetrate tissues due to absorption by biomolecules and optical scattering. While visible light with a shorter wavelength typically penetrates up to a few millimeters of tissue, the efficient penetration depth of near-IR (800 nm) is up to approximately 2 cm. Ideally, photoactivation should require irradiation in the spectral range of the so-called ‘therapeutic window’ of 650–900 nm with the highest penetration depth of soft human tissues [13,17].

As the amount of light is limited by tissue-dependent absorption, it is important that the photoswitching quantum yield is sufficient to be triggered by the number of photons that can reach the target site.

Moreover, photoconversion needs to be sufficient to generate meaningful differences in activity. Additionally, the thermal stability of the active form, which can be in the range of picoseconds to years, should be suitable for the intended application [18,19].

### 2.2. Pharmacological Properties

When introducing a molecular photoswitch into a drug, identification of the appropriate modification site is fundamental, as alterations of structurally optimized drugs can lead to a significant change in the pharmacokinetic and pharmacodynamic properties [13,16].

As the structural modification can lead to a loss of activity, the ideal is to develop a photoswitchable analogue that, in one photoisomeric state, retains the potency level of the original compound, but for which photoisomerization leads to a significant and reversible potency change [16].

It has to be considered that the altered structure also influences the polarity and consequently the diffusion of the drug. For pharmaceutical applications, it is additionally important to ensure that both photoisomers are sufficiently water-soluble and metabolically stable, and that they do not show general toxicity at the applied concentration [16].

## 3. Photoswitchable Microtubule-targeting Agents

### 3.1. Combretastatin A-4

The stilbene derivative combretastatin A-4 (**CA-4**) is a natural product isolated from the stem wood of the South African tree *combretum caffrum* [20]. It is a microtubule-destabilizing agent, similar in structure and function to colchicine. In addition, it was found that both compounds occupy similar space in the same binding site in β-tubulin [21].

**CA-4** possesses anti-cancer activity against multiple human cancer cell lines. In particular, its vascular disrupting properties and its avoidance of multidrug-resistance make **CA-4** and its derivatives attractive drug candidates for tumor chemotherapy [22].

As a stilbene derivative it can photoisomerize and it was found that the thermodynamically less stable *Z*-**CA-4** is 60-fold more potent than the *E*-isomer [21]. However, *E → Z* photoisomerization of stilbenes requires irradiation with toxic UV light (<300 nm) and is not completely reversible due to the photochemical 6π electrocyclization of *Z*-stilbene [23]. While these characteristics prevent the qualification of **CA-4** as a photopharmacological agent, its light-dependent activity gave rise to the development of numerous photoswitchable analogues.

### 3.2. Combretastatin A-4 Analogues: Azobenzenes

**Figure 4:**
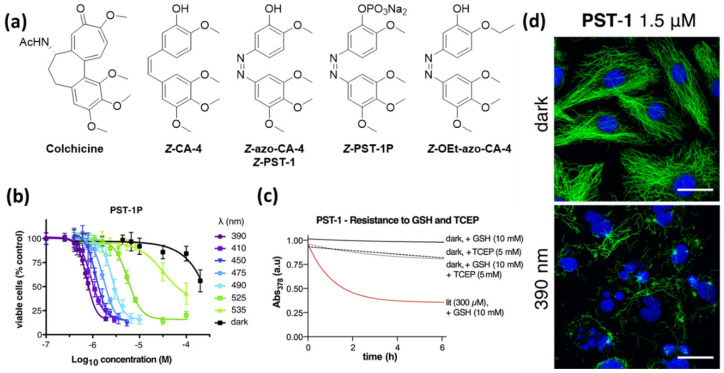
(**a**) Photoswitchable azologues of Combretastatin A-4 (**CA-4**) discussed in this review. (**b**) Viability assays demonstrate the light-tunable potency of **PST-1P**. (**c**) *E*-**PST-1** is stable in presence of GSH but degraded by the reducing agent TCEP. The corresponding photoisomer *Z*-**PST-1** degrades rapidly in presence of GSH. (**d**) **PST-1** causes breakdown of microtubules and nuclear fragmentation selectively under irradiation with 390 nm. Treatment with the same concentration but in the dark does not affect microtubule structure. (MDA-MB-231 cells; α-tubulin (green), DNA (blue); scale bars, 20 mm) [24,28]. (**b**,**d**): Reprinted with permission from the ref. [24]. Copyright (2015) Elsevier. (**c**): Reprinted with permission from the ref. [24,28]. Copyright (2021) Elsevier.

The first photopharmacological azologues of **CA-4,** in which the C=C double bond is replaced by an isosteric N=N double bond, have been reported in three independent studies by Borowiak et al. (photostatins, **PST**s) [24], Engdahl et al. (azo-combretastatin A4, **azo-CA-4**) [25], and Sheldon et al. [26], as well as – shortly after – by Rastogi et al. [27]. The key advantages of azobenzenes compared to stilbenes are the insignificant number of side-reactions during photoisomerization and the extensive research that has already been conducted on multiple modifications of the scaffold that enable tuning of its photophysical properties. Irradiation of the biologically inactive *E*-isomers of these azologues with low-intensity visible light leads to the formation of the colchicine-like *Z*-isomers with microtubule-destabilizing activity (Figure 4).

Borowiak et al. synthesized a series of azologues with different substitution patterns as well as azalogues of known **CA-4** prodrugs. They identified compound **azo-CA-4** and its prodrug **PST-1P**, which additionally bears a phosphate group, as the most promising candidates [24]. **Azo-CA-4** was also synthesized and investigated by the two other groups, and Sheldon et al. performed computational studies to support the assumed similarities in the structures and natural charges of **azo-CA-4** and **CA-4** [26]. The reversible photoswitching in aqueous medium (PBS Buffer) and the photostability of the compounds over many switching cycles were demonstrated. *E* → *Z* isomerization can be induced by irradiation with violet light (380–420 nm) yielding up to 90% *Z*-isomer, while green light irradiation (500–530 nm) gives approximately 85% *E*-isomer [24,25]. Whereas Borowiak et al. determined for *Z*-**azo-CA-4** a half-live of 6.2 min, Sheldon et al. found it to be considerably longer with 85 min under the same conditions (20% acetonitrile in PBS at 37 °C) [26]. In an independent study, Rastogi et al. measured a half-life of 46 h in methanol [27].

All three groups demonstrated a decrease in overall cytotoxicity compared to **CA-4** and a significantly enhanced potency upon irradiation with light in the range 380–410 nm. This was demonstrated for HeLa cells cervical cancer [24,25], breast adenocarcinoma epithelial cell line MDA-MB-231 cells [24,26] and human umbilical vein endothelial cells (HUVECs) [26].

Borowiak et al. additionally found that the dose–response curves obtained after treatment with **PST-1P** (HeLa, MTT) at a series of wavelengths correlate to the *E*/*Z* ratios at these respective wavelengths, which demonstrates the tunability of the compound’s activity (Figure 4) [24]. Sheldon et al. found that the cytotoxicity can also be tuned by varying the irradiation pulse frequency during the incubation time of the cells. An increased pause between the pulses led to a decrease in toxicity [26].

In tubulin polymerization assays, all three groups showed that, as with **CA-4,** irradiated **azo-CA-4** is also a potent inhibitor of tubulin polymerization, while the non-irradiated compound behaves similarly to the negative control [24,25,26].

Borowiak et al. also performed a light-dependent cell-cycle analysis and found that irradiated **azo-CA-4** leads to G_2_/M arrest (MDA-MB-231 cells/ HeLa/ HEK293T cells), which is a typical observation for microtubule-targeting agents. Furthermore, they demonstrated dynamic photocontrol with a rescue protocol, which shows that photoisomerization is possible in cellulo without degradation [24].

In in vivo experiments with *C. elegans* embryos, Borowiak et al. demonstrated that, after treatment with **azo-CA-4,** mitotic progression can be blocked in metaphase by irradiation with blue light. It is possible to target single cells while neighboring cells continue mitosis, and this mitotic arrest can be reversed by irradiation with green light [24].

In recent years, **azo-CA-4** has already been applied as a tool to study microtubule organization and function in early mouse embryos [29] and tissue morphogenesis in Drosophila [30], as well as to investigate biological processes such as spinal cord regeneration in zebrafish [31].

In 2017 Rastogi et al. published an **CA-4** azologue (**OEt-azo-CA-4**) with improved photopharmacological properties compared to **azo-CA-4** where one of the methoxy groups is replaced by an ethoxy group. This compound possesses an allover greater potency and a greater difference in activity between non-irradiated and irradiated samples (600-fold greater activity in the presence of UV light for HeLa cells). The results of docking studies showed that the binding conformations of compounds **azo-CA-4** and **OEt-azo-CA-4** were almost identical and also overlap with the structure of bound combretastatin obtained via X-ray crystallography. These results suggest that **azo-CA-4** as well as **OEt-azo-CA-4** bind to the colchicine binding site. However, they did not identify a significant difference between the two azologues that could explain their different bioactivities. Rastogi et al. proposed that the prolonged half-life of *Z*-**OEt-azo-CA-4** compared to **azo-CA-4** could promote the greater lit/dark ratio of the EC_50_ values [27].

Wranik et al. recently reported that they could follow the release of **azo-CA-4** from the colchicine binding pocket in tubulin caused by light-triggered *Z → E* isomerization via time-resolved serial crystallography. It was found that the position and interactions of **azo-CA-4** in the binding pocket resemble that of **CA-4** very closely and they were able to follow rearrangements within the binding pocket after the isomerization that led to a release of *E*-**azo-CA-4** within 10 ms [32].

Although **azo-CA-4** has proven to be useful, it also has its limitations. All research groups working with azobenzene-derived antimitotic agents observed a significantly lower potency of the compounds when compared to **CA-4**. This can be explained by the generally observed metabolic instability of electron-rich azobenzenes, caused by cellular glutathione. Sheldon et al. [26] as well as Gao et al. [28] found that the degradation of the bioactive *Z*-**azo-CA-4** formed under irradiation proceeds even faster than that of the corresponding *E*-isomer. This significantly limits the applicability of azobenzene-based photopharmacological agents not only because of the loss in potency but also the formation of potentially harmful metabolites.

### 3.3. Combretastatin A-4 Analogues: Hemithioindigos

Hemithioindigo (HTI) is an emerging molecular photoswitch [33,34,35] that has already found numerous applications in molecular motors [36,37,38], tweezers [39] and in the photomodulation of biological systems [40,41]. To expand the toolbox of photochromic antimitotic agents to other photoswitchable motifs and consequently parameters such as the tolerance of chemical substituents, Sailer et al. designed HTI-based tubulin inhibitors. They embedded the structural pattern known from **CA-4**—the combination of a trimethoxyphenyl ring and a methoxyphenyl ring—into the HTI framework. The resulting compounds were found to be stable in the presence of glutathione and also to tolerate functionalization with tautomerizable polar functional groups including *para*-hydroxy substituents [42].

**Figure 5:**
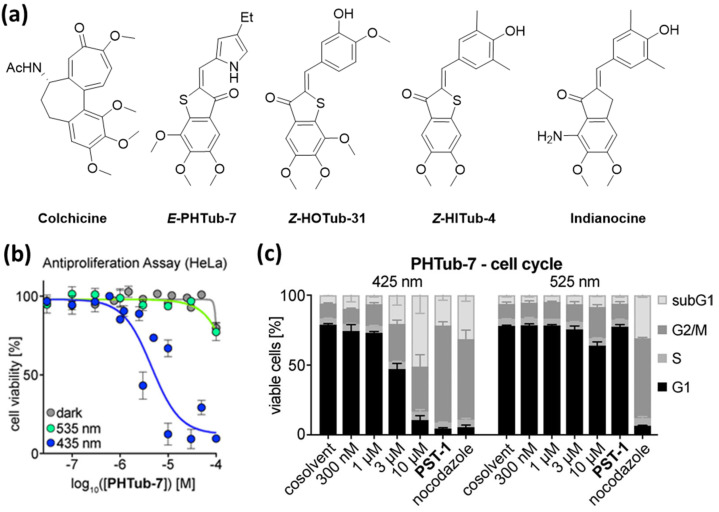
(**a**) The design of hemithioindigo-based photoswitchable microtubule destabilizing agents was based on colchicine and indianocine. (**b**) **PHTub-7** shows light-dependent antiproliferative effects in cell viability assays. (**c**) Cell cycle analysis after treatment with **PHTub-7** show significant G_2_/M arrest and cell death selectively under 425 nm but not under 525 nm irradiation. Reprinted with permission from the ref [44]. Copyright (2021) John Wiley and Sons.

After evaluating the photopharmacological properties of compounds bearing various combinations of substituents on the trimethoxyphenyl ring, located either on the hemistilbene side or on the thioindigo half, they identified two derivatives with a significant difference in antimitotic potencies between the dark and the irradiated state (HeLa, MTT). Interestingly, their lead compound (**HOTub-31**, Figure 5) showed a fourfold lower EC_50_ upon irradiation, which is contrary to the lit-state bioactivity observed for **azo-CA-4**. The less active metastable *E*-isomer switches back spontaneously with a half-life of ca. 30 min at 37 °C in a DMSO/PBS (3:1) mix. The light-dependent disintegration of the microtubule network structure was demonstrated by fluorescence microscopy. Additionally, in flow cytometry experiments, it was found that, while treatment with **HOTub-31** in darkness led to G_2_/M arrest (3 µM, dark), this effect was not observed for samples irradiated with 450 nm [42].

The significant loss in potency when compared to **CA-4** and **azo-CA-4** can be explained by differences in the structural parameters including the longer end-to-end distance in the scaffold, and the smaller torsion angel when compared to colchicine, **CA-4** or **azo-CA-4**. Such differences limit the applicability of HTIs as mimics of these pharmacophores.

To enhance the potency of HTI-based microtubule-targeting agents, Sailer et al. altered the main framework and designed compounds based on indanocine, a microtubule inhibitor that also binds to the colchicine binding site and has a molecular scaffold more similar to HTIs. They identified HTI-based antimitotic agents with an enhanced potency compared to the previously reported HOTubs. Active compounds were ca. one order of magnitude less active than indanocine and exhibited 2.2–4.1-fold enhanced potency in the dark compared to samples irradiated with 450 nm. As in previous studies, the mechanistic assessment was performed by detecting tubulin polymerization inhibition cell-free assays and visualization of disruption of microtubule network via immunofluorescence staining as well as confocal microscopy. Furthermore, light-dependent bioactivity with G_2_/M phase arrest was verified by flow cytometry [43].

### 3.4. Combretastatin A-4 Analogues: Pyrrole Hemithioindigos

In subsequent studies, Sailer et al. implemented near-quantitative photoswitchable pyrrole HPIs to address two additional factors that limit the photoswitchability of azobenzenes in biological assays: the limited bidirectional photoisomerization within the biologically compatible light spectrum and the restricted accessibility of high photostationary states due to fixed-wavelength lasers available in confocal microscopes [44].

As with the previously reported photoswitchable microtubule-targeting agents, the designed PHTubs bear steric analogies to colchicine and were designed to enable the reversible control of cell viability and microtubule dynamics with visible light. PHTubs showed a good photoswitchability within the visible light spectrum (ca. 90% *E*-isomer at 430–450 nm vs. 95% *Z*-isomer at 515–530 nm in MeCN) and were found to be stable under reducing conditions (glutathione) and repeated photoisomerization. In the first antiproliferation assays with HeLa and Jurkat cells, **PHTub-7** was identified as the lead compound, with an over 14-fold enhanced potency upon activation with 450 nm (Jurkat cell line). The tubulin-specificity was confirmed with immunostaining imaging as well as polymerization inhibition assays with purified tubulin and light-dependent G_2_/M phase arrest detected via flow-cytometry (Figure 5). Remarkably, they also demonstrated that during confocal microscopy this compound can be photoactivated in live cells with spatial and temporal precision. Whereas microtubule dynamics were not significantly influenced by the addition of the *Z*-isomer, irradiation with 442 nm laser pulses resulted in suppressed microtubule dynamics [44].

### 3.5. Combretastatin A-4 Analogues: Heterostilbenes

**Figure 6:**
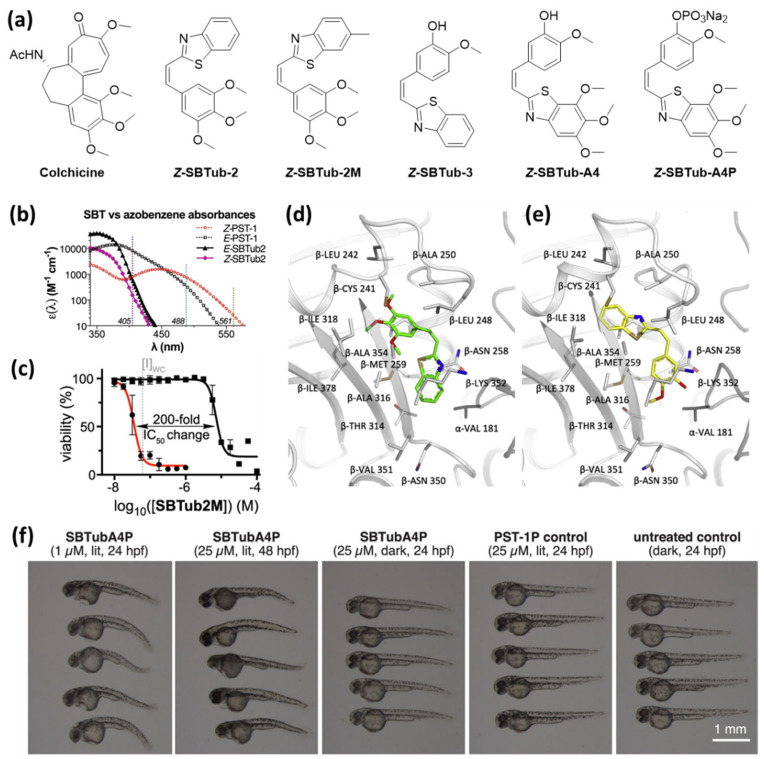
(**a**) Heterostilbenes with photoswitchable antimitotic activity. (**b**) Comparison of absorbance spectra of both photoisomers of **SBTub2** and **PST-1** (**azo-CA-4**) demonstrates orthogonality to the common laser wavelengths above 410 nm applied for imaging. (**c**) The results of light-dependent viability assays with **SBTub2M** show highly nonlinear dose–response profiles with a high lit/dark ratio of bioactivity. (**d**,**e**) X-ray crystal structure analysis of the complexes (**d**) tubulin-*Z*-SBTub2 (PDB:6ZWC) and (**e**) tubulin-*Z*-SBTub3 (PDB: 6ZWB) revealed that both compounds bind to the colchicine site. (**f**) **SBTubA4P** (1 or 25 μM) causes morphological abnormalities in the development of *D. rerio* selectively in the lit state [28,45]. (**c**,**f**): Reprinted from the ref. [45]; (**b**,**d**,**e**): Reprinted with permission from the ref. [24,28]. Copyright (2021) Elsevier.

Whereas PHTubs switch with wavelengths used for imaging with confocal microscopes, SBTubes photoisomerize with light frequencies that are orthogonal to wavelengths used to excite green fluorescent protein (GFP), which is commonly used as a fluorescent label in biological systems (Figure 6b). This feature prevents undesired SBTub photoswitching caused by the lasers during imaging processes. Additionally, they are stable in the presence of cytoplasmic glutathione and the metastable *Z*-isomers show a significantly enhanced half-life that enable long-term effects without prolonged irradiation [28].

Styrylbenzothiazole (SBT) contains a photoswitchable C=C double bond. The two photoisomers are similar in their geometry to *E*- and *Z*-stilbene, but in case of SBT, the irreversible photochemical electrocyclization—a typical side reaction of stilbenes—is blocked [46,47]. The absorption spectrum of SBT is shifted bathochromically relative to the stilbene system. This enables photoswitching with the frequencies of 365–410 nm (UV and near-UV light), which can be used in the cellular context. At the same time, the absorption of both photoisomers above 410 nm is negligible, which prevents unwanted photoisomerization during imaging experiments with the common 488 nm lasers used for GFP imaging.

Two of the demonstrated SBT derivatives enabled photoactivation of antiproliferative effects on the HeLa cervical cancer cell line with an over 20-fold enhanced potency upon irradiation with 360 nm. Light-dependent microtubule inhibition was confirmed in cell-free tubulin polymerization assay and using flow cytometric analysis (G_2_/M phase arrest selectively upon irradiation). Additionally, the authors successfully crystallized complexes of *Z*-**SBTub2** and *Z*-**SBTub3** with the tubulin-DARP in D1, and crystal structure analysis revealed that both compounds bind to the colchicine binding site. They also found that, with **SBTub3,** microtubule polymerization can be controlled even with subcellular precision. After treating primary rat hippocampal neurons with **SBTub3**, they were able to selectively block microtubule polymerization of irradiated (405 nm) neurites, without affecting non-irradiated neighboring neurites [28].

SBTubs especially stand out because of the high thermal stability of the *Z*-isomers and the metabolic stability of both photoisomers. However, in combination with the very low band separation between the two photoisomers, this results in a system that only allows irreversible light-triggered activation. Low solubility in aqueous media and the overall potency reduction are two potential areas of improvement for practical applicability of these systems.

This motivated Gao et al. to synthesize a second library of colchicine-inspired SBT compounds, designed for multichannel imaging without affecting the photoswitchable system. Through structure–activity studies, they identified two compounds, **SBTubA4** and **SBTub2M,** with an enhanced potency in the mid-nanomolar range and a 30–200-fold photomodulation in their antimitotic activity [45]. Furthermore, they designed a fully water-soluble prodrug **SBTubA4P** suitable for in vivo applications. Additional attempts to enable reversible photoactivation by accelerating the thermal relaxation of the *Z*-isomers by introducing electron-donating substituents were not successful. These optimized SBTubs also function as photoactivatable (*E* → *Z* isomerization at 360 to ca. <440 nm) tubulin inhibitors that bind to the colchicine binding site with high thermal and metabolic stability. Gao et al. demonstrated an enhanced range of applications for **SBTubA4P** as a photoactivatable antimitotic agent with single cell precision in 2D cell cultures and 3D organoids, as well as in small tissue explants and early-stage animals (zebrafish, clawed frog). The orthogonality to the GFP imaging channels enables two-channel protein imaging in parallel to spatiotemporally localized photocontrol of microtubule dynamics by illumination with the common 405 nm laser. Although the photophysical properties of the SBTubs do not allow thermal or light-triggered deactivation of the compounds, the authors observed that, after photoactivation of **SBTubA4P,** microtubule dynamics recovered with a half-life of ca. 20 s in a 2D cell culture and ca. 10 min in organoids and in vivo. The authors suggest that this reversibility is caused by diffusion of the bioactive *Z*-isomer out of the irradiated area. Overall, this system enabled reversible and spatiotemporally localized inhibition of mitotic progression in zebrafish *D. rerio* in repeatable photoactivation-recovery cycles.

### 3.6. Combretastatin A4 Analogues: Spiropyrans

**Figure 7:**
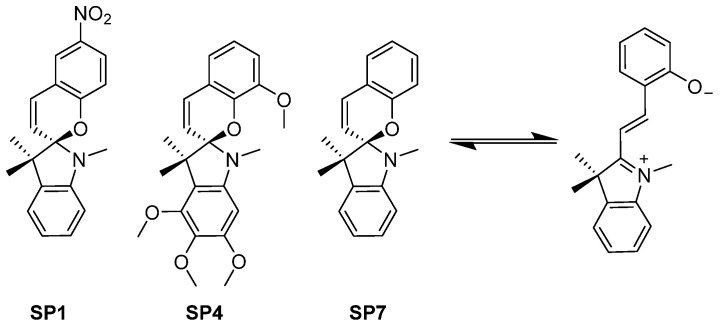
Spiropyrane derivatives that can be photoisomerized with UV-light in an aqueous environment reported by Rastogi et al. [48].

In 2020, Rastogi et al. reported a series of spiropyrans designed as colchicine analogues that are switchable with UV-light in aqueous solutions (5% DMSO in PBS) to the respective open-ring merocyanine forms (Figure 7).

They identified two compounds, **SP1** and **SP4,** with an over 10-fold enhanced bioactivity upon irradiation with micromolar EC_50_ values and weak inhibitory activities towards tubulin dynamics, determined in cell-free assays. Unfortunately, the derivative **SP7** identified as the most potent inhibitor of cell growth with the highest activity as a tubulin polymerization inhibitor did not exhibit meaningful bioactivity photomodulation [48].

### 3.7. Paclitaxel- and Epothilone-Based Agents

To expand the spectrum of applications of microtubule-targeting pharmaceuticals, paclitaxel- and epothilone-based photoswitchable microtubule-stabilizing agents have also been developed. These compounds complement the set of microtubule-targeting photopharmacological agents with another spectrum of biological effects and differ in pharmacological properties and stoichiometry [49].

A. Müller-Deku et al. synthesized a library of 3′-azobenzamide-taxanes by varying the relative position of the azo bond to the amide, and found that the orientation of the diazene in *meta* position to the amide led to the smallest loss in overall potency and enabled the highest dark/lit ratio of EC_50_ values. They additionally installed methoxy or dimethylamino groups in different positions on the azobenzene moiety to tune the composition of the photostationary states as well as the thermal half-life of the respective *E*-isomers and the solubility of the compounds. The introduction of methoxy groups led to an increased dynamic range of *Z*-isomer photoswitchability from 3-fold to 9-fold. Whereas the thermal relaxation of the respective *Z*-isomers of unsubstituted and methoxylated compounds was in the range of hours to days, the half-life the *para*-amino derivatives was too short to be determined. The results of light-dependent cell viability tests with HeLa cells of all compounds led to the identification of **AzTax3MP** as the lead compound with a bioactivity profile comparable with docetaxel but with lower potency. It acts as a microtubule-stabilizing agent with the activity increasing upon irradiation in cell-free tubulin polymerization assays and in light-dependent immunofluorescence imaging. A twofold increase in the G_2_/M-arrest level was observed using flow cytometry upon exposure of the **AzTax3MP** to light. Müller-Deku et al. moreover reported that **AzTax3MP** can be applied to reversibly photomodulate microtubule networks and microtubule-dependent functions in cancer (HeLa) and neuronal (rat primary hippocampal neurons) cells with high temporal resolution and on a single-cell down to subcellular precision [49].

Analogous to the expansion of photoswitchable scaffolds in microtubule-destabilizing agents, Gao et al. attempted to further improve photoswitchable paclitaxel-based agents (SBTax) by introducing styrylbenzothiazole (SBT). Contrary to the anticipation, the resulting compound proved to be unsuitable due to its poor solubility combined with a very low potency and dynamic range of bioactivity photomodulation. Gao et al. reasoned that the taxanes’ intrinsic characteristics—including the high molecular weight and low solubility—limit its applicability in the development of photoswitchable analogues [50].

After having found that SBTax is not suitable as a photopharmacological agent, Gao et al. chose an alternative design based on epothilones, which also bind to the taxane binding site but are structurally simpler and superior in solubility as well as in potency. They additionally altered the photoswitchable motif and exchanged SBT by the smaller styrylthiazole (ST) scaffold to minimize the size and increase the solubility of the resulting compounds. Epothilone B and D analogues were synthesized, and two designs were tested by keeping the thiazole ring at its original position or inverting the photoswitch by connecting the switch via a phenyl ring to the macrolactone. As with SBTs, the designed STEpos can be efficiently isomerized to thermally stable *Z*-isomers with near-UV light. However, in contrast to SBTs, photodegradation was observed upon irradiation < 340 nm. As in the case of SBT-based microtubule-targeting agents, the limiting factor of STEpos is the irreversibility of the light-induced effect due to the high thermal stability of the *Z*-isomers in combination with the negligible absorption band separation between the two photoisomers. For all compounds, a good photoswitchability of bioactivity was observed in antiproliferation assays. Unlike the other derivatives, **STEpo4** was found to be less active, which is an interesting observation; however, due to the irreversibility of the light-induced deactivation, this derivative was less suitable for practical applications. Under irradiation, light-activated **STEpo2** was identified as the lead compound with a comparably high lit/dark ratio of the EC_50_ values and satisfactory synthetic accessibility. The assumed mode of action as a microtubule-stabilizing agent was confirmed by cell-free tubulin polymerization assays and by immunofluorescence imaging of treated cells. The mixture obtained upon irradiation with 360 nm incubation with low concentrations of **STEpo2** led to defective mitotic spindles. Additionally, at high concentrations, the formation of microtubule bundles was observed. Furthermore, the authors demonstrated that **STEpo2** enables in situ suppression of microtubule dynamics upon 405 nm pulsing during continuous imaging of GFP-labelled cells using the 487 nm laser [50].

### 3.8. Others

In 2019, Imperatore et al. reported two chiral microtubule-targeting benzodiazo *N*-substituted pyrrole derivatives with an enhanced potency upon irradiation with blue light. They performed antiproliferation assays on HCT-116 p53-/- cancer cells in the dark and under irradiation with 435 nm, respectively. Interestingly, they observed a synergistic effect when combining the two diastereomers. The mixture was significantly more potent than each of the isolated isomers and also exhibited greater photoswitchable activity. In cell-free tubulin polymerization assays, however, the authors found that only one of the isomers (**1RS**) acts as a microtubule-destabilizing agent, with an enhanced activity upon irradiation. A lower activity was determined for the diastereomeric mixture and no change in tubulin polymerization was observed when **1RR** was added [51].

## 4. Conclusions

Various photoswitchable microtubule-targeting compounds have been designed and applied to spatiotemporally control microtubule dynamics with light. The earliest derivatives were **CA-4** azologues, first reported in 2015, and already implemented as tools to study microtubule dynamics. The limitations of the electron-rich azobenzene scaffold, such as its metabolic instability, elicited studies on alternative photoswitchable motifs. Spiropyrans, hemithioindigos, pyrrole-hemithioindigos, styrylbenzothiazoles and styrylthiazoles have been implemented successfully and enable reversible photocontrol of cell growth.

All of these derivatives have unique benefits but also limitations. Currently there is no compound available that combines reversible photoswitchability, high potency, a high photomodulation of bioactivity and metabolic stability. Additionally, so far, no microtubule-targeting compound has been reported that can be photoisomerized within the phototherapeutic window, which would allow for targeting of solid tumors inside of the human body.

## References

[1] Dumontet C., Jordan M.A. (2010). Microtubule-binding agents: A dynamic field of cancer therapeutics. Nat. Rev. Drug Discov..

[2] Steinmetz M.O., Prota A.E. (2018). Microtubule-Targeting Agents: Strategies To Hijack the Cytoskeleton. Trends Cell Biol..

[3] Akhmanova A., Steinmetz M.O. (2008). Tracking the ends: A dynamic protein network controls the fate of microtubule tips. Nat. Rev. Mol. Cell Biol..

[4] Nogales E. (2000). Structural insights into microtubule function. Annu. Rev. Biochem..

[5] Luduena R.F. (2013). A Hypothesis on the Origin and Evolution of Tubulin. Int. Rev. Cell Mol. Biol..

[6] Fees C.P., Moore J.K. (2018). Regulation of microtubule dynamic instability by the carboxy-terminal tail of β-tubulin. Life Sci. Alliance.

[7] Mitchison T., Kirschner M. (1984). Dynamic Instability of Microtubule Growth. Nature.

[8] van Haren J., Wittmann T. (2019). Microtubule Plus End Dynamics—Do We Know How Microtubules Grow?: Cells boost microtubule growth by promoting distinct structural transitions at growing microtubule ends. Bioessays.

[9] Downing K.H. (2000). Structural basis for the interaction of tubulin with proteins and drugs that affect microtubule dynamics. Annu. Rev. Cell Dev. Biol..

[10] Brouhard G.J., Rice L.M. (2014). The contribution of alpha beta-tubulin curvature to microtubule dynamics. J. Cell Biol..

[11] Borys F., Tobiasz P., Poterala M., Krawczyk H. (2021). Development of novel derivatives of stilbene and macrocyclic compounds as potent of anti-microtubule factors. Biomed. Pharmacother..

[12] Peterson J.R., Mitchison T.J. (2002). Small molecules, big impact: A history of chemical inhibitors and the cytoskeleton. Chem. Biol..

[13] Sharma M., Friedman S.H. (2021). The Issue of Tissue: Approaches and Challenges to the Light Control of Drug Activity. ChemPhotoChem.

[14] Redmond R.W., Kochevar I.E. (2006). Spatially resolved cellular responses to singlet oxygen. Photochem. Photobiol..

[15] Klan P., Solomek T., Bochet C.G., Blanc A., Givens R., Rubina M., Popik V., Kostikov A., Wirz J. (2013). Photoremovable Protecting Groups in Chemistry and Biology: Reaction Mechanisms and Efficacy. Chem. Rev..

[16] Velema W.A., Szymanski W., Feringa B.L. (2014). Photopharmacology: Beyond Proof of Principle. J. Am. Chem. Soc..

[17] Lerch M.M., Hansen M.J., van Dam G.M., Szymanski W., Feringa B.L. (2016). Emerging Targets in Photopharmacology. Angew. Chem. Int. Ed..

[18] Broichhagen J., Frank J.A., Trauner D. (2015). A Roadmap to Success in Photopharmacology. Acc. Chem. Res..

[19] Fuchter M.J. (2020). On the Promise of Photopharmacology Using Photoswitches: A Medicinal Chemist’s Perspective. J. Med. Chem..

[20] Pettit G.R., Singh S.B., Boyd M.R., Hamel E., Pettit R.K., Schmidt J.M., Hogan F. (1995). Antineoplastic Agents. 291. Isolation and Synthesis of Combretastatins A-4, A-5, and A-6. J. Med. Chem..

[21] Gaspari R., Prota A.E., Bargsten K., Cavalli A., Steinmetz M.O. (2017). Structural Basis of cis- and trans-Combretastatin Binding to Tubulin. Chem.

[22] Tron G.C., Pirali T., Sorba G., Pagliai F., Busacca S., Genazzani A.A. (2006). Medicinal chemistry of combretastatin A4: Present and future directions. J. Med. Chem..

[23] Pianowski Z.L. (2019). Recent Implementations of Molecular Photoswitches into Smart Materials and Biological Systems. Chemistry.

[24] Borowiak M., Nahaboo W., Reynders M., Nekolla K., Jalinot P., Hasserodt J., Rehberg M., Delattre M., Zahler S., Vollmar A. (2015). Photoswitchable Inhibitors of Microtubule Dynamics Optically Control Mitosis and Cell Death. Cell.

[25] Engdahl A.J., Torres E.A., Lock S.E., Engdahl T.B., Mertz P.S., Streu C.N. (2015). Synthesis, Characterization, and Bioactivity of the Photoisomerizable Tubulin Polymerization Inhibitor azo-Combretastatin A4. Org. Lett..

[26] Sheldon J.E., Dcona M.M., Lyons C.E., Hackett J.C., Hartman M.C.T. (2016). Photoswitchable anticancer activity via trans–cis isomerization of a combretastatin A-4 analog. Org. Biomol. Chem..

[27] Rastogi S.K., Zhao Z.Z., Barrett S.L., Shelton S.D., Zafferani M., Anderson H.E., Blumenthal M.O., Jones L.R., Wang L., Li X.P. (2018). Photoresponsive azo-combretastatin A-4 analogues. Eur. J. Med. Chem..

[28] Gao L., Meiring J.C.M., Kraus Y., Wranik M., Weinert T., Pritzl S.D., Bingham R., Ntouliou E., Jansen K.I., Olieric N. (2021). A Robust, GFP-Orthogonal Photoswitchable Inhibitor Scaffold Extends Optical Control over the Microtubule Cytoskeleton. Cell Chem. Biol..

[29] Zenker J., White M.D., Templin R.M., Parton R.G., Thorn-Seshold O., Bissiere S., Plachta N. (2017). A microtubule-organizing center directing intracellular transport in the early mouse embryo. Science.

[30] Singh A., Saha T., Begemann I., Ricker A., Nüsse H., Thorn-Seshold O., Klingauf J., Galic M., Matis M. (2018). Polarized microtubule dynamics directs cell mechanics and coordinates forces during epithelial morphogenesis. Nat. Cell Biol..

[31] Vandestadt C., Vanwalleghem G.C., Castillo H.A., Li M., Schulze K., Khabooshan M., Don E., Anko M.-L., Scott E.K., Kaslin J. (2019). Early migration of precursor neurons initiates cellular and functional regeneration after spinal cord injury in zebrafish. bioRxiv.

[32] Wranik M., Weinert T., Slavov C., Masini T., Furrer A., Gaillard N., Gioia D., Ferrarotti M., James D., Glover H. (2022). Molecular snapshots of drug release from tubulin over eleven orders of magnitude in time. bioRxiv.

[33] Kink F., Collado M.P., Wiedbrauk S., Mayer P., Dube H. (2017). Bistable Photoswitching of Hemithioindigo with Green and Red Light: Entry Point to Advanced Molecular Digital Information Processing. Chemistry.

[34] Petermayer C., Dube H. (2018). Indigoid Photoswitches: Visible Light Responsive Molecular Tools. Acc. Chem. Res..

[35] Hoffmann K., Guentner M., Mayer P., Dube H. (2019). Symmetric and nonsymmetric bis-hemithioindigos—Precise visible light controlled shape-shifters. Org. Chem. Front..

[36] Guentner M., Schildhauer M., Thumser S., Mayer P., Stephenson D., Mayer P.J., Dube H. (2015). Sunlight-powered kHz rotation of a hemithioindigo-based molecular motor. Nat. Commun..

[37] Grill K., Dube H. (2020). Supramolecular Relay-Control of Organocatalysis with a Hemithioindigo-Based Molecular Motor. J. Am. Chem. Soc..

[38] Bach N.N., Josef V., Maid H., Dube H. (2022). Active Mechanical Threading by a Molecular Motor. Angew. Chem. Int. Ed..

[39] Wiedbrauk S., Bartelmann T., Thumser S., Mayer P., Dube H. (2018). Simultaneous complementary photoswitching of hemithioindigo tweezers for dynamic guest relocalization. Nat. Commun..

[40] Kitzig S., Thilemann M., Cordes T., Ruck-Braun K. (2016). Light-Switchable Peptides with a Hemithioindigo Unit: Peptide Design, Photochromism, and Optical Spectroscopy. ChemPhysChem.

[41] Zhang L., Linden G., Vazquez O. (2019). In search of visible-light photoresponsive peptide nucleic acids (PNAs) for reversible control of DNA hybridization. Beilstein J. Org. Chem..

[42] Sailer A., Ermer F., Kraus Y., Lutter F.H., Donau C., Bremerich M., Ahlfeld J., Thorn-Seshold O. (2019). Hemithioindigos for Cellular Photopharmacology: Desymmetrised Molecular Switch Scaffolds Enabling Design Control over the Isomer-Dependency of Potent Antimitotic Bioactivity. Chembiochem.

[43] Sailer A., Ermer F., Kraus Y., Bingham R., Lutter F.H., Ahlfeld J., Thorn-Seshold O. (2020). Potent hemithioindigo-based antimitotics photocontrol the microtubule cytoskeleton in cellulo. Beilstein J. Org. Chem..

[44] Sailer A., Meiring J.C.M., Heise C., Pettersson L.N., Akhmanova A., Thorn-Seshold J., Thorn-Seshold O. (2021). Pyrrole Hemithioindigo Antimitotics with Near-Quantitative Bidirectional Photoswitching that Photocontrol Cellular Microtubule Dynamics with Single-Cell Precision*. Angew. Chem. Int. Ed..

[45] Gao L., Meiring J.C.M., Varady A., Ruider I.E., Heise C., Wranik M., Velasco C.D., Taylor J.A., Terni B., Weinert T. (2022). In Vivo Photocontrol of Microtubule Dynamics and Integrity, Migration and Mitosis, by the Potent GFP-Imaging-Compatible Photoswitchable Reagents SBTubA4P and SBTub2M. J. Am. Chem. Soc..

[46] Mishra A., Thangamani A., Chatterjee S., Chipem F.A., Krishnamoorthy G. (2013). Photoisomerization of trans-2-[4′-(dimethylamino)styryl]benzothiazole. Photochem. Photobiol..

[47] El-Hendawy M.M., Fayed T.A., Awad M.K., English N.J., Etaiw S.E.H., Zaki A.B. (2015). Photophysics, photochemistry and thermal stability of diarylethene-containing benzothiazolium species. J. Photochem. Photobiol. A.

[48] Rastogi S.K., Dunnigan J.K., Towne A.C., Zhao Z.Z., Du L.Q., Brittain W.J. (2021). Photopharmacology of Azo-Combretastatin-A4: Utilizing Tubulin Polymerization Inhibitors and Green Chemistry as the Key Steps. Curr. Org. Chem..

[49] Müller-Deku A., Meiring J.C.M., Loy K., Kraus Y., Heise C., Bingham R., Jansen K.I., Qu X., Bartolini F., Kapitein L.C. (2020). Photoswitchable paclitaxel-based microtubule stabilisers allow optical control over the microtubule cytoskeleton. Nat. Commun..

[50] Gao L., Meiring J.C.M., Heise C., Rai A., Muller-Deku A., Akhmanova A., Thorn-Seshold J., Thorn-Seshold O. (2022). Photoswitchable Epothilone-Based Microtubule Stabilisers Allow GFP-Imaging-Compatible, Optical Control over the Microtubule Cytoskeleton. Angew. Chem. Int. Ed..

[51] Imperatore C., Scuotto M., Valadan M., Rivieccio E., Saide A., Russo A., Altucci C., Menna M., Ramunno A., Mayol L. (2019). Photo-control of cancer cell growth by benzodiazo N-substituted pyrrole derivatives. J. Photochem. Photobiol. A.

